# Spinal oncologic paraparesis: Analysis of neurological and surgical outcomes in patients with intramedullary, extramedullary, and extradural tumors

**DOI:** 10.3389/fonc.2022.1003084

**Published:** 2023-01-04

**Authors:** Obada T. Alhalabi, Stefan Heene, Vincent Landré, Jan-Oliver Neumann, Moritz Scherer, Basem Ishak, Karl Kiening, Klaus Zweckberger, Andreas W. Unterberg, Alexander Younsi

**Affiliations:** ^1^ Department of Neurosurgery, Heidelberg University Hospital, Heidelberg, Germany; ^2^ Department of Neurosurgery, City Hospital of Brunswick, Brunswick, Germany

**Keywords:** paraparesis, spinal metastases, spinal ependymomas, intramedullary tumors, McCormick score

## Abstract

**Objectives:**

Paraparesis due to oncologic lesions of the spine warrants swift neurosurgical intervention to prevent permanent disability and hence maintain independence of affected patients. Clinical parameters that predict a favorable outcome after surgical intervention could aid decision-making in emergency situations.

**Methods:**

Patients who underwent surgical intervention for paraparesis (grade of muscle strength <5 according to the British Medical Research Council grading system) secondary to spinal neoplasms between 2006 and 2020 were included in a single-center retrospective analysis. Pre- and postoperative clinical data were collected. The neurological status was assessed using the modified McCormick Disability Scale (mMcC) Score. In a univariate analysis, patients with favorable (discharge mMcC improved or stable at <3) and non-favorable outcome (discharge mMcC deteriorated or stable at >2) and different tumor anatomical compartments were statistically compared.

**Results:**

117 patients with oncologic paraparesis pertaining to intramedullary lesions (n=17, 15%), intradural extramedullary (n=24, 21%) and extradural lesions (n=76, 65%) with a mean age of 65.3 ± 14.6 years were included in the analysis. Thoracic tumors were the most common (77%), followed by lumbar and cervical tumors (13% and 12%, respectively). Surgery was performed within a mean of 36±60 hours of admission across all tumors and included decompression over a median of 2 segments (IQR:1-3) and mostly subtotal tumor resection (n=83, 71%). Surgical and medical complications were documented in 9% (n=11) and 7% (n=8) of cases, respectively. The median hospital length-of-stay was 9 (7-13) days. Upon discharge, the median mMcC score had improved from 3 to 2 (p<0.0001). At last follow-up (median 180; IQR 51-1080 days), patients showed an improvement in their mean Karnofsky Performance Score (KPS) from 51.7±18.8% to 65.3±20.4% (p<0.001). Localization in the intramedullary compartment, a high preoperative mMcC score, in addition to bladder and bowel dysfunction were associated with a non-favorable outcome (p<0.001).

**Conclusion:**

The data presented on patients with spinal oncologic paraparesis provide a risk-benefit narrative that favors surgical intervention across all etiologies. At the same time, they outline clinical factors that confer a less-favorable outcome like intramedullary tumor localization, a high McCormick score and/or bladder and bowel abnormalities at admission.

## Introduction

Spinal space-occupying lesions of suspected oncologic etiology can be classified based on magnetic resonance imaging (MRI) into intra- or extradural (1). Intradural tumors are mostly benign (2) and can be further divided into intra- and extramedullary lesions (3), representing 10-15% of all primary. tumors of the central nervous system. In contrast, extradural metastases with potential spinal cord compression (MSCC) possess the lion share of spinal tumors (60%) (4) and affect about 5% of all cancer patients (5), with intramedullary metastases known to be rare (6, 7).

Lesions with spinal cord compression usually cause a myelopathy resulting a variety of symptoms in affected patients, including backpain, sensory and motor deficits, in addition to gait abnormalities (8, 9). Depending on localization and size, further clinical signs like radiculopathy or impaired bladder and bowel function can become apparent (10). Consequently, para- or even tetraparesis can occur with a subsequent loss of ambulation and a significant reduction of quality of life (11, 12).

Early decompression surgery is warranted in affected patients to preserve their neurological outcome (13, 14). To this end, a wide range of surgical techniques is implemented including posterior decompression and stabilization, posterior decompression without stabilization, and posterior decompression with gross total resection (GTR), especially for benign tumors (15), or subtotal tumor resection (2, 16). Adjuvant radiotherapy and chemotherapy remain viable options for patients with malignant primary spinal tumors and metastases and sub-totally resected or recurring benign tumors (17–20).

Previous studies have shown different clinical variables to correlate with a good neurological recovery and a viable postoperative ambulatory status in patients with spinal oncologic paraparesis, including a good pre-operative neurological status and a preserved bowel function in MSCC (21). As another important factor the time interval between symptom onset and treatment has been discussed (18). Interestingly, similar predictors were determined for intradural tumors, adding age and localization (22, 23), although fewer studies have reported on such compared to MSCC, possibly due to their lower incidence (24). However, even though previous works have individually highlighted the role of surgery in improving the neurological outcome of patients with one single etiology of spinal oncologic paraparesis, a simultaneous report on all possible etiologies and a comparison of clinical parameters between them in one study have not yet been performed.

This study hence aims to evaluate the role of surgical intervention in improving the neurological outcome of patients with spinal oncologic paraparesis of different etiologies (intramedullary, extramedullary intradural, and extradural) in one original work. By comparing the clinical presentation, management, and outcome of affected patients in a comprehensive neurosurgical center, we consolidate findings from several previous studies on patients by highlighting differences in the presentation and prognosis of such tumors based on clinical variables and anatomical localization.

## Methods

In this retrospective study, clinical, histopathological, and radiological data of patients admitted to a single neurosurgical center between 2006 and 2020 for surgical treatment of oncologic paraparesis (grade of muscle strength < 5 according to the British Medical Research Council (BMRC) grading system in at least one muscle group in both lower extremities) pertaining to intraspinal neoplasms in different anatomical compartments (intramedullary, intradural extramedullary or extradural) between 2004 und 2017 were collected. The pre- and postoperative neurological status was assessed using the modified McCormick Disability Scale Score (mMcC) and the Frankel Grade (FG). To include the oncologic aspect of these patients, the Karnofsky Performance Scale (KPS) was also assessed. American Society of Anesthesiologists (ASA) scores were extracted from medical records (assigned by the anesthesiologist responsible for the general anesthesia of the respective patient). The extent of tumor resection was documented as gross total resection (GTR), subtotal resection (STR) or biopsy. Additional follow-up data was collected where available. Favorable outcome at discharge was defined as an improvement in the mMcC score and non-favorable outcome as a postoperative deterioration. In case of a stable mMcC score, only values <3 were regarded as a favorable outcome, whereas an mMcC >2 was assigned a non-favorable outcome.

### Statistical analysis

Patient characteristics were analyzed using descriptive statistics. Continuous variables are reported as mean ± standard deviation or median (and interquartile range (IQR)), while ordinal and nominal variables are presented as numbers and frequencies. Missing data are designated as such. Comparison of nominal variables between groups was performed using Chi-Square or Fischer’s exact test (depending on group size). Mann–Whitney tests were used for non-parametric and double-tailed student’s T-test (in paired-samples and independent samples) or analyses of variance (ANOVA) for parametric, normally distributed data. Significance was deemed to be reached at p<0.05 and all statistical analyses were performed using SPSS (version 21, IBM, Armonk, New York) and Graphpad PRISM (Version 7).

## Results

### Patient characteristics and clinical presentation

A total of 117 patients were included in this study with a median age of 66.7(56-75) years. The patient population comprised 73 males (62%). MRI scans of the spine were available in 113 of the 117 cases (96%), with 114 lesions showing contrast enhancement (97%). Tumors arose in three different anatomical compartments: extradural (n=76, 65%), intradural extramedullary (n=24, 21%) and intramedullary in 17 patients (15%). All extradural tumors were metastases, mostly arising from prostate (n=25, 21%) and lung cancer (n=17, 15%). The remaining 34 metastases where of breast (5%), gastro-intestinal tract (4%), head and neck (4%) and renal cancer (3%) or of unknown origin (5%). The intradural extramedullary tumors were pre-dominantly meningiomas (19 of 24 extramedullary tumors) with three cases of neurinomas and neurofibroma in two cases. The intramedullary glial tumors consisted of ependymomas in 8 cases (7%) and astrocytomas in 9 cases (9%) as histological diagnoses (**Table 1**).

**Table 1:** Patient demographics and imaging findings.

**Characteristics**	**Values**
**Number of patients**	117
**Gender (%)**	
Male	72 (62%)
Female	45 (38%)
**Age**	
median, IQR	66.7, 56-75 years
mean±SD	65.3 ± 14.6 years
**Tumors (%)**	
**Intradural Intramedullary**	17 (15%)
Ependymoma	8 (7%)
Astrocytoma, including glioblastoma	9 (8%)
**Intradural Extramedullary**	24 (21%)
Meningioma	19(16%)
Other*	5 (4%)
**Extradural Metastases - origin**	76 (65%)
Prostate	25 (21%)
Lung	17 (15%)
Breast	6 (5%)
GI-tract	5 (4%)
Head and Neck	5 (4%)
Renal cancer	4 (3%)
Others	6 (5%)
Unknown	6 (5%)
**ASA-Score at surgery (%)**	
ASA <3	31(26%)
ASA >2	86 (74%)
**Location of tumors (%)**	
Cervical	13 (12%)
Thoracic	90 (77%)
Lumbar	14 (12%)
**Spinal levels affected (%)**	
**1**	43 (37%)
**2**	40 (34%)
**3**	26 (22%)
**4+**	6 (5%)

IQR, Inter Quartile Range; SD, Standard Deviation; ASA, American Society of Anesthesiology. *Other tumors include Neurinoma (n=3) and neurofibroma (n=2). Percentages displayed are calculated from the total patient number (n=117).

In terms of localization, 77% percent of the tumors (n=90) were found in the thoracic spine, with the remaining lesions almost equally split between the cervical (n=13) and lumbar (n=14) region. Cervical tumors were mostly intramedullary (7 out of all 13 cervical cases) whereas lumbar tumors were mostly located extradural (8 out of all 14 lumbar tumors). In most patients (n=74, 63%), more than one spinal level was affected, but only in 5% (n=6), the tumor extended over more than four levels.

The major chief complaints of patients prior to admission were motor deficits (as in, paresis, n=51, 44%) and subsequently gait abnormalities (n=28, 24%). The median time between onset of the paresis and presentation was 5 (2-28) days. Further symptoms included back pain (n=17, 15%), sensory deficits (n=14, 12%) and bladder dysfunction (n=6, 5%). Upon clinical examination, according to the inclusion criteria, all patients showed a motor deficit in both legs, with 75% (n=84) suffering from severe paraparesis (grade of muscle strength ≤ 3 according to the BMRC) in their ‘best’ muscle group and three patients (3%) experiencing complete paraplegia. Sensory deficits were very common as well (n=97, 83%) and bladder and bowel dysfunction were documented in 57% (n=66) and 25% (n=32) of cases, respectively. The median mMcC of all patients upon admission was 3 (3 - 4), with 32% of patients showing an mMcC of 4 (n=38), most patients had an FG of C (n=60, 51%) and the mean KPS was 51.7±18.8% (**Table 2**).

**Table 2:** Comparison of patients with favorable (n=70) and non-favorable (n=47) outcomes.

Characteristic	All	Favorable outcome	Non-favorable outcome	p-value
Number of patients	117	70	47	
Age				
**median, IQR**	66.7, 56-75 years	70.0, 59-76 years	65.7, 51-74 years	0.09^1^
**mean±SD**	65.3 ± 14.6 years	67.2±13.9 years	62.49±15.9 years	
**First symptom (%) Paresis**	51 (44%)	26 (37%)	25 (53%)	0.79^2^
**Gait abnormality**	28 (24%)	22 (31%)	6 (13%)	
**Back Pain**	17 (15%)	11 (16%)	6 (13%)	
**Sensory deficit**	14 (12%)	6 (9%)	8 (17%)	
**Bowl/bladder dysfunction**	6 (5%)	4 (6%)	2 (4%)	
**KPS on admission median, IQR**	40, 40-70 %	60, 40-77.5 %	40, 30-50 %	**<0.0001^1^**
**mean±SD**	51.7±18.8 %	57.4±19.3 %	43.2±14.2 %	
McCormick Score (mMcC) on admission (%)				
**2**	18 (15%)	16 (23%)	2 (4%)	**0.007^2^**
**3**	61 (52%)	37 (53%)	24 (51%)	
**4**	38 (32%)	17 (24%)	21 (45%)	
**Median mMcC, IQR**	3, 3-4	3, 3-3.25	3, 3-4	**0.0026^3^**
**Frankel Grade (FG) on admission (%) Grade A**	16 (14%)	5 (7%)	11 (23%)	**0.007^2^**
**Grade B**	22 (19%)	12 (17%)	10 (21%)	
**Grade C**	60 (52%)	37 (53%)	24 (51%)	
**Grade D**	18 (16%)	16 (23%)	2 (4%)	
**Symptoms on admission (%) Paresis**	117(100%)^a^	70 (100%)	47 (100%)	
**Back pain**	41 (35%)	25 (36%)	16 (34%)	
**Radiating Pain**	14 (12%)	9 (13%)	5 (11%)	0.853^2^
**Sensory deficit**	97 (83%)	59 (84%)	38 (81%)	0.717^2^
**Bladder dysfunction**	66 (57%)	34 (49%)	32 (68%)	0.492^2^
**Bowel dysfunction**	32 (28%)	11 (16%)	21 (45%)	0.037^2^
**Duration of paresis median, IQR**	5, 2-28 days	7, 2-30 days	4, 1-21 days	**<0.001^2^**
**mean±SD**	80±265 days	79.8±168 days	80.9±366 days	0.983^2^
Degree of worst paresis (%)^b^				
**> Grade 3/5 BMRC in one muscle group**	12 (11%)	11 (12%)	4 (9%)	0.100^2^
**< Grade 4/5 BMRC in one muscle group**	100 (89%)	59 (88%)	41 (91%)	
Degree of best paresis (%)^b^				
**> Grade 3/5 BMRC in one muscle group**	28 (25%)	24 (31%)	7 (16%)	0.088^2^
**< Grade 4/5 BMRC in one muscle group**	84 (75%)	46 (69%)	38 (84%)	
**Anatomical compartment of tumors (%) intramedullary**	17 (15%)	6 (9%)	11 (24%)	**<0.001^2^**
**extramedullary (including extradural)**	100 (85%)	64 (91%)	36 (76%)	**0.026^2^**
**intradural**	24 (21%)	22 (31%)	2 (4%)	0.170^2^
**extradural Metastases**	76 (65%)	41(60%)	34 (72 %)	
Location of tumors (%)				
**Cervical spine**	13 (12%)	6 (8%)	7 (15%)	0.552^2^
**Thoracic spine**	90 (77%)	55 (79%)	35 (74%)	
**Lumbar spine**	14 (12%)	9 (13%)	5 (11%)	
**ASA score (%)**				
**< ASA 3**	31(26%)	21(30%)	10 (22%)	0.295^2^
**> ASA 2**	86 (74%)	49 (70%)	37 (78%)	
**Time to surgery after admission median, IQR (days)**	1, 0-1	1, 0-1	1, 0-2	0.980^2^
**mean±SD (days)**	1.53±2.54	1.57±2.65 days	1.47±2.4	
Levels of laminectomy^c^				
**1**	36 (31%)	21 (30%)	15 (32%)	0.568^2^
**2**	47 (40%)	29 (41%)	18 (38%)	
**3**	27 (23%)	18 (26%)	9 (19%)	
**4+**	6 (5%)	2 (2%)	4 (8%)	
**median, IQR**	2, 1-3	2, 1-3	2, 1-3	0.8482^1^
**Tumor resection GTR**	31 (26%)	24 (34%)	7 (15%)	0.075^2^
**STR**	83 (71%)	45 (64%)	38 (81%)	
**Biopsy**	2 (2%)	1 (1%)	1 (2%)	
**Peri-operative use of corticosteroids**	**80 (68%)**	**45 (64%)**	**35 (74%)**	**0.04^2^**
**Use of Neuromonitoring**	**13 (11%)**	**9 (13%)**	**4 (9%)**	**0.463^2^**
**Dorsal stabilization**	9 (8%)	4 (6%)	5 (11%)	0.334^2^
**Duration of surgery median, IQR (min)**	145, 118-218	150, 120-218	140, 110-210	
**mean±SD (min)**	173±81.1	171±74.6	176±91.1	0.740^1^
**Surgical complications (%)**	11 (9%)	6 (9%)	5 (11%)	0.707^2^
**Postoperative spinal hematoma**	5 (4%)	3 (3%)	2 (2%)	
**Wound healing disorders**	3 (3%)	2 (2%)	1 (1%)	
**CSF fistula**	1 (1%)	0 (0%)	1 (1%)	
**Other**	2 (2%)	1 (1%)	1 (1%)	
**Revision surgery (%)**	8 (7%)	5 (7%)	3 (6%)	0.873^2^
**Medical complications (%)**	13 (11%)	4 (6%)	9 (19%)	0.023^2^
Hospital length of stay median, IQR (days)		9, 7-13	8, 6-14	0.2823^1^
mean±SD (days)	10±5.9	10±5.4	10±6.6	
KPS on discharge				
**median, IQR**	60, 50-70 %	70, 60-80 %	50, 40-50 %	
**mean±SD**	59.4±18.8 %	69.1±15.3 %	44.9±10.6 %	**0.001^1^**
**McCormick Score on discharge (%)**				**0.001^2^**
**1**	3 (3%)	3 (4%)	0(0%)	
**2**	60 (51%)	60 (86%)	0(0%)	
**3**	31 (26%)	7 (10%)	24(51%)	
**4**	23 (20%)	0 (0%)	23(49%)	
**Median mMcC, IQR**	2, 2-3	2, 2-2	3, 3-4	**<0.0001** ^3^
**FG on discharge (%)**				**<0.001** ^2^
**Grade A**	12 (10%)	0 (0%)	12 (26%)	
**Grade B**	11 (9%)	0 (0%)	11 (23%)	
**Grade C**	31 (26%)	7 (10%)	24 (51%)	
**Grade D**	60 (51%)	60 (86%)	0 (0%)	
**Grade E**	3 (3%)	3 (4%)	0 (0%)	

SD, Standard Deviation; IQR, Inter Quartile Range; BMRC, British Medical Research Council grading system; FG, Frankel Grade; KPS, Karnofsky Performance Scale, ASA, American Society of Anesthesiologists; ^1^ Student’s t-test; ^2^ Chi-Squared-test. **^3^** Mann Whitney test. ^a^3 patients presented with a complete paraplegia; ^b^In 5 patients, no documented degree of paresis was found. ^C^In one case, a hemilaminectomy was performed and in another a laminoplasty. Percentages displayed are calculated from the total number of patients in each column.

### Surgical treatment and clinical course

The median time between admission and neurosurgical intervention was 1 (0-1) days. Corticosteroids were prescribed to 68% (n=80) of patients perioperatively. A laminectomy as means of spinal decompression and/or access to intradural tumors was performed on one spinal level in 31% of the cases (n=36), two levels in 40% (n=47) and three levels in 23% (n=27). Six patients (5%) received laminectomies on four spinal levels with additional dorsal instrumentation. Electrophysiological neuromonitoring (NM) was performed during 11% (n=13) of surgeries, comprising patients with intramedullary and intradural tumors. A laminotomy on one spinal level with a laminoplasty was only performed in one case (<1%). In terms of tumor debulking, gross total resection was achieved in 31 patients (26%), whereas most patients (n=83, 71%) only received subtotal resection. Mere biopsies were acquired in two cases (2%) only. The median surgery time was 145 (118-218) minutes.

Surgical complications occurred in 11 patients (9%) and comprised postoperative spinal hematoma (n=5, 4%), wound healing disorders (n=3, 3%), one CSF fistula, intraoperative air embolism and postoperative hydrocephalus (n=1, 1% each). Of those, all except three cases required revision surgery (n=8, 7%). With 74% (n=86) of the patients included in this cohort having an ASA score of ≥ 3 or more, the rate of medical complications was 11% (n=13), including urinary tract infections (n=2, 2%), postoperative pulmonary embolism, deep vein thrombosis, myocardial infarction, and death with unknown cause (n=1, 1% each). The median hospital length-of-stay of all patients was 9 (7-13) days.

Upon discharge, 55 patients (47%) showed an improvement of their mMcC score and in further 59 patients (51%), the mMcC score remained unchanged. Of those, 15 (13%) had a discharge mMcC score of < 3 and 44 (38%) a mMcC score of > 2. In three patients (3%), the postoperative mMC score deteriorated. Hence, after surgical treatment of spinal oncologic paraparesis, 70 patients (60%) had improved their mMcC or preserved mild deficits (favorable outcome), while 47 (40%) patients remained with severe postoperative motor deficits (non-favorable outcome). In terms of the FG, similar effects were observed. Indeed, the proportion of patients with FG D and E (useful motor function) increased from 16% (n=18) to 54% (n=63) postoperatively. This improvement was translated into a better postoperative mean KPS (59.4±18.1%) at discharge as well. Moreover, relief of the chief symptom was achieved in 73 patients (62%).

At the last follow-up after a median of 147 (51-1080) days, clinical data was available for 66 patients (56%) and neurological data was available for 44 patients (38%). During that period, a total of 61 (52%) patients, mostly patients with metastases (n=52) and primary intramedullary tumors (n=8), had received adjuvant therapy.

### Favorable and non-favorable outcomes: a comparative analysis

Firstly, we compared patients with favorable and non-favorable outcomes after surgical treatment of spinal oncologic paraparesis to assess potential clinical parameters that might influence their functional status at discharge (**Table 2**). Patients presenting with a lower preoperative mMcC score and FG at admission (indicative of a less sever preoperative disability) had a significantly better postoperative outcome (p=0.007). In line with this, patients with a favorable outcome also showed a higher KPS at admission (mean KPS 57.4% vs 43.2% in the non-favorable outcome group, p<0.001). Moreover, the presence of bowel dysfunction before surgery was significantly associated with worse outcomes (p=0.007). Of note, the proportion of patients with intramedullary tumors was larger in the non-favorable outcome group, while intradural extramedullary tumors highly enriched in the favorable outcome group (p<0.001).

Due to this observation, a second analysis looking into differences between tumors in the distinct anatomical compartments was performed. It could be shown that extramedullary tumors in general exhibited a better postoperative neurological outcome compared to intramedullary tumors as reflected by a higher mMcC score and FG (D+E in 83% vs. 35%, p=0.033, Chi-Square test) at discharge. Prior to surgery, only 16% of this patient cohort had a preserved ambulatory function (FG D+E), which was increased up to 54% postoperatively, restoring ambulatory status in 45 patients (38%), with extramedullary and extradural tumor patients showing higher rates of restored walking ability than patients with intramedullary tumors (p=0.033, **Table 3**).

**Table 3:** Comparison of patients with different compartment localizations of tumors.

Characteristic	All	Intramedullary	Extramedullary intradural	Extradural	
**Number of patients (%)**	117 (100%)	17 (15%)	24 (21%)	76 (65%)	n. a.
**Age (years)** mean±SD	65.3 ± 14.6	56.4±18.5	67.5±14.9	66.9±13.3	**0.0221**
**First symptom (%)**					**<0.001^2^**
Paresis	51 (44%)	3 (3%)	2 (2%)	46 (39%)	
Gait abnormality	27 (23%)	5 (4%)	17 (15%)	5 (4%)	
Back pain	16 (14%)	2 (2%)	2 (2%)	12 (10%)	
Sensory deficit	14 (12%)	5 (4%)	2 (2%)	7 (6%)	
Bowl/bladder dysfunction	7 (6%)	2 (2%)	1 (1%)	4 (3%)	
**KPS on admission mean±SD**	51.7±18.8%	63.53±16.6%	74.6±8.3%	41.6±12.8%	**<0.001^1^**
**McCormick Score on admission (%)**					**<0.001^2^**
**2**	18 (15%)	5 (4%)	11 (9%)	2 (2%)	
**3**	60 (51%)	7 (6%)	8 (7%)	45 (38%)	
**4**	38 (32%)	5 (4%)	5 (4%)	28 (24%)	
**FG on admission (%)**					**<0.001^2^**
**Grade A**	16 (14%)	1 (1%)	0 (0%)	15 (13%)	
**Grade B**	22 (19%)	4 (3%)	5 (4%)	13 (11%)	
**Grade C**	60 (51%)	7 (53%)	8 (53%)	45 (38%)	
**Grade D**	18 (15%)	5 (4%)	11 (9%)	2 (2%)	
Symptoms on admission (%)					
**Paresis**	117 (100%)	17 (15%)	24 (21%)	76 (65%)	117 (100%)
**Back pain**	41 (35%)	3 (3%)	6 (5%)	32 (27%)	0.0742
**Radiating Pain**	14 (12%)	4 (3%)	1 (1%)	9 (8%)	0.1722
**Sensory deficit**	96 (82%)	10 (9%)	23 (20%)	63 (54%)	**0.0082**
**Bladder dysfunction**	66 (56%)	10 (9%)	8 (7%)	48 (41%)	**0.032**
**Bowel dysfunction**	32 (27%)	5 (4%)	5 (4%)	22 (19%)	0.6912
**Duration of paresis mean±SD**	80±265 days	268±603 days	180±213 days	7±12 days	**<0.001^1^**
**Location of tumors (%)**					**<0.001^2^**
**Cervical spine**	13 (11%)	7 (6%)	5 (4%)	1 (1%)	
**Thoracic spine**	89 (76%)	7 (6%)	16 (14%)	66 (56%)	
**Lumbar spine**	14 (12%)	3 (3%)	3(3%)	8 (7%)	
**ASA score (%)**					**<0.001^2^**
**< ASA 3**	30 (26%)	8 (7%)	11 (9%)	11 (9%)	
**> ASA 2**	86 (74%)	9 (8%)	13 (11%)	64 (55%)	
**Time to surgery after admission mean±SD (days)**	1.53±2.54	2.29±2.14	2.83±3.96	0.95±1.79	**0.002^1^**
**Levels of laminectomy^c^**					**0.068^2^**
**1**	36 (31%)	2 (2%)	4 (3%)	30 (26%)	
**2**	46 (39%)	6 (5%)	16 (14%)	24 (21%)	
**3**	27 (23%)	7 (6%)	4 (3%)	16 (14%)	**0.026^3^**
**4+**	6 (5%)	2 (2%)	0 (0%)	4 (3%)	
**median, IQR**	2, 1-3	3, 2-3	2, 2	2, 1-3	**<0.001^2^**
**Tumor resection**					
**GTR**	30 (26%)	6 (5%)	21 (18%)	3 (3%)	
**STR**	83 (71%)	10 (9%)	2 (2%)	71 (61%)	
**Biopsy**	2 (2%)	1 (1%)	1 (1%)	0 (0%)	0.006**^2^**
**Peri-operative use of corticosteroids**	**80 (68%)**	**16 (14%)**	**12 (10%)**	**52 (44%)**	**<0.001^2^**
**Use of Neuromonitoring**	**13 (11%)**	**7 (6%)**	**6 (5%)**	**0 (0%)**	0.186^2^
**Dorsal stabilization**	9 (8%)	0 (0%)	0 (0%)	9 (8%)	0.098^1^
**Duration of surgery mean±SD (min)**	173±81.1	262±82.6	205±82.5	140±57.4	0.295^2^
**Surgical complications (%)**	11 (9%)	4 (3%)	2 (2%)	5 (4%)	
**Postoperative epidural hematoma**	4 (3%)	1 (1%)	1 (1%)	2 (2%)	
**Wound healing disorders**	2 (2%)	1 (1%)	1 (1%)	0 (0%)	
**CSF fistula**	1 (1%)	0 (0%)	0 (0%)	1 (1%)	
**Other**	4 (3%)	2 (1%)	0 (0%)	2 (1%)	**0.008^2^**
**Revision surgery (%)**	8 (7%)	4 (3%)	2 (2%)	2 (2%)	**0.023^2^**
**Medical complications (%)** **Hospital length of stay**	13 (11%)	4 (3%)	5 (6%)	4 (3%)	**0.022^1^**
**mean±SD (days)**	10.3±5.9	12.9±7.6	11.8±5.9	9.20±5.2	**<0.001^1^**
**KPS on discharge mean±SD**	59.4±18.8%	59.4±16.0%	77.5±15.3%	53.6±16.0%	**0.021^2^**
McCormick Score on discharge (%)					
**1**	3 (3%)	0 (0%)	0 (0%)	3 (3%)	
**2**	60 (51%)	6 (5%)	20 (17%)	34 (29%)	
**3**	30 (26%)	5 (10%)	3 (10%)	22 (51%)	
**4**	23 (20%)	6 (0%)	1 (0%)	16 (49%)	**0.033^2^**
**FG on discharge (%) Grade A**	12 (10%)	2 (0%)	1 (0%)	9 (26%)	
**Grade B**	11 (9%)	4 (0%)	0 (0%)	7 (23%)	
**Grade C**	30 (26%)	5 (4%)	3 (3%)	22 (19%)	
**Grade D**	60 (51%)	6 (5%)	20 (17%)	34 (29%)	
**Grade E**	3 (3%)	0 (0%)	0 (0%)	3 (3%)	**<0.001^2^**
Outcome on discharge					
**Favorable**	70 (60%)	6 (5%)	22 (19%)	42 (36%)	
**Non-favorable**	46 (39%)	11 (9%)	2 (2%)	33 (28%)	
**Follow-up**	66 (56%)	12 (10%)	13 (11%)	41 (35%)	
**Available patients**	547±735	1088±978	1071±749	223±403	
Mean time till last follow-up ± SD (days)					
**Median time till last follow-up (IQR) (days)**	147(29-1050)	993(114-1729)	1076(512-1302)	52 (17-218)	
**Patients receiving adjuvant therapy**	61(52%)	8(7%)	1(1%)	52 (44%)	

SD, Standard Deviation; FG, Frankel Grade; KPS, Karnofsky Performance Scale, ASA, American Society of Anesthesiologists; IQR, Interquartile range. ^1^ANOVA (analysis of variance); ^2^ Chi-Squared-test. ^3^Kruskal-Wallis test. ^a^3 patients presented with a complete paraplegia; ^b^In 5 patients, no documented degree of paresis was found. ^C^ In one case, a hemilaminectomy was performed and in another a laminotomy. Percentages represent a fraction of the whole patient cohort included in the study.

Interestingly, tumor localization in terms of the affected spinal level did not significantly affect outcome, although most cervical tumors were intramedullary and most lumbar tumor were extradural (p<0.001), nor did the ‘number’ of laminectomy levels across the three different anatomical compartments (**Table 2** and **3**). There was a trend towards better outcomes in patients with gross total vs. subtotal resection, however this did not reach statistical significance (**Table 2**). GTR was mostly achieved in intradural extramedullary tumors, whereas STR was more reserved to intramedullary and extradural tumors (p<0.001, **Table 3**).

The use of peri-operative corticosteroids did not positively influence functional outcome. Indeed, patients receiving corticosteroids were over-proportionally represented in the non-favorable cohort (p<0.04, **Table 2**). However, a compartment-wise analysis of corticosteroid-usage revealed higher rates of application in patients with intramedullary and extradural tumors, which inherently show worse outcomes (**Table 3**). A separate analysis of corticosteroid use in the subgroup of patients with extradural metastases (n=75) showed no difference in outcome (favorable in 58% (n=30/52) of the patients with vs. 50% (n=11/22) without corticosteroid use, p=0.5429, Chi-Square test). Across all patients, the use of electrophysiological neuromonitoring (NM - MEPs: Motor evoked potentials and SSEPs: Somatosensory evoked potentials) did not yield a better outcome in this study (**Table 2**). When analyzing the subgroup of patients with relevant use of neuromonitoring in clinical routine (intradural tumors, n=42), no differences were observed in terms of outcome (n=9/28 patients (32%) with favorable outcome received neuromonitoring vs. n=4/14 patients (29%) with non-favorable outcome, p>0.99, Fisher’s exact test).

**Figure 1:**
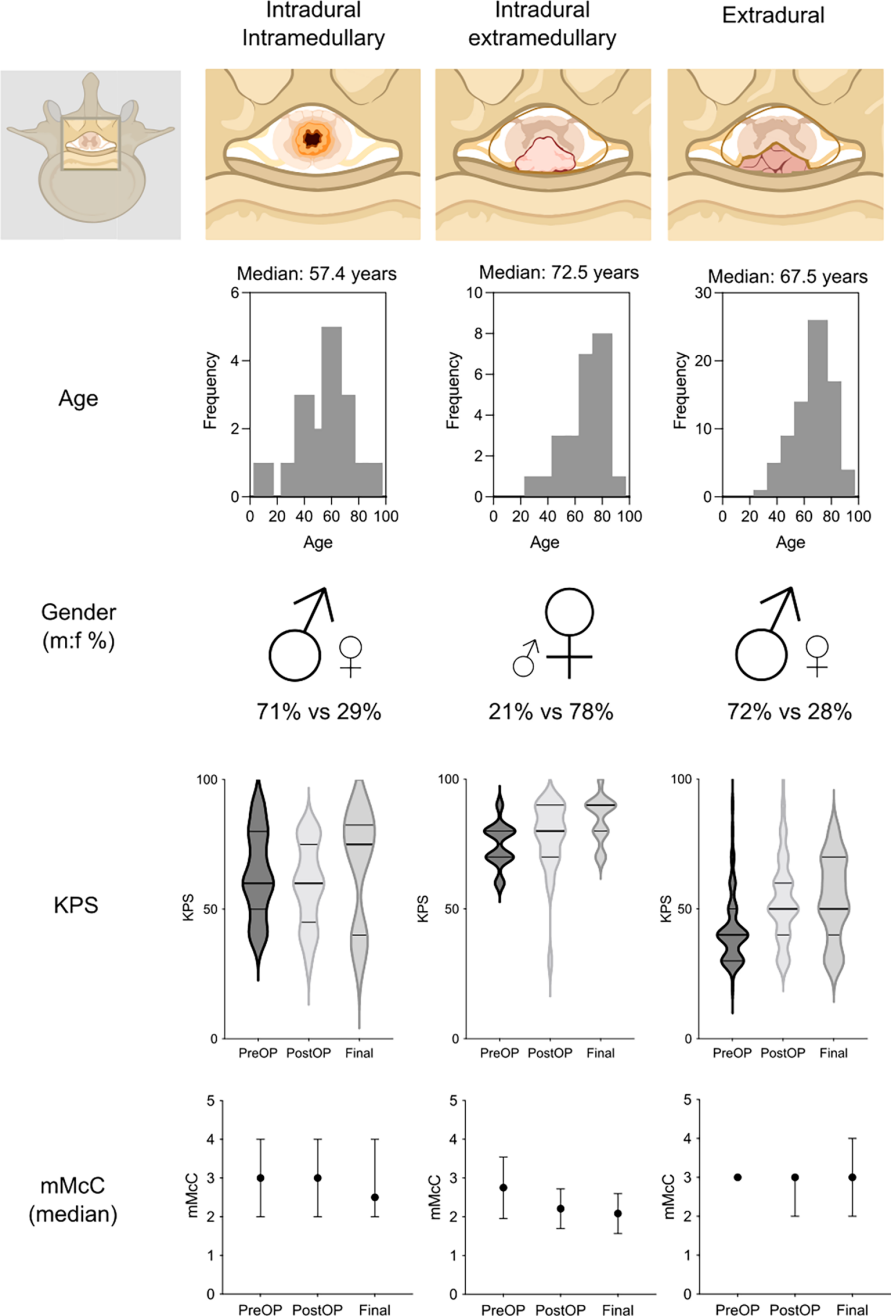
Schematic comparison of patient characteristics and their functional outcome at admission vs. discharge or final follow-up in patients with intramedullary, intradural extramedullary and extradural tumors. KPS, Karnofsky Performance Scale; mMcC, modified McCormick Disability Scale Score. Parts of the figure were created with BioRender.com.

Complication-wise, there were no relevant differences in surgical complications neither in the favorable vs. non-favorable outcome nor in the anatomical compartment-based analysis (p=0.707 and p=0.295, respectively). However, patients with a non-favorable outcome showed significantly higher occurrence of medical complications (6% vs. 19%, p=0.023). This was particularly true for patients with intramedullary tumors (p=0.023), although the extradural metastases’ cohort showed proportionally more morbid patients (ASA ≥ 3 in 84% of patients with extradural tumors compared to 47% in patients with intramedullary tumors, p<0.001). **Figure 1** provides a comparative summary of the main differences in outcomes between the three different anatomical compartments.

### Postoperative and long-term outcomes of oncologic paraparesis patients

To provide a ‘bigger picture’ of postoperative and long-term outcomes of all patients included, we performed pre-post analyses of KPS and mMcC across the different patient subgroups.

For all patients in the study cohort, a significant postoperative improvement was noted, both in KPS (51.7% to 59.4%, p<0.001, paired student’s t-test) and in mMcC (median mMcC score from 3 at admission to 2 at discharge, p<0.001, Wilcoxon matched-pairs signed rank test, **Table 4**). This improvement was more pronounced in patients with favorable outcome (KPS 57.4, vs. 69.1%, p<0.001 and median mMcC score from 3 at admission to 2 at discharge, p<0.001), and virtually non-existent in patients with unfavorable outcome (KPS 43.2% vs. 44.9%, p=0.272 and median mMcC score from 3 at admission to 3 at discharge, p=0.250).

**Table 4:** Comparison of functional parameters at admission vs. discharge or follow-up in patients with favorable outcome, non-favorable outcome, intramedullary, intradural extramedullary and extradural tumors.

Characteristic	Admission	Discharge	p-value	Follow-up*	p-value
KPS (mean±SD)	n=117	n=117	Admission vs discharge	n=45	Admission vs follow-up
All patients (n=117)	51.7±18.8	59.4±18.1	**<**0.001^1^	65.3±20.4	<0.001^1^
Favorable outcome (n=70)	57.4±19.4	69.1±15.3	<0.001^1^	69.4±18.7	<0.001^1^
Non-favorable outcome (n=47)	43.2±14.2	44.9±10.6	0.2721	51.3±20.2	11
Intramedullary tumors (n=17)	63.5±16.6	59.4±16.0	0.168	67.0±22.1	0.5401
Intradural extramedullary tumors (n=24)	74.6±8.30	77.5±13.9	0.3801	85.0±9.10	<0.001^1^
Extradural tumors (n=76)	41.6±12.8	53.6±16.0	<0.001^1^	54.4±15.9	0.0611
**Median McCormick score, IQR**					
All patients (n=117)	3, 3-4	2, 2-3	<0.001^2^	3, 2-3.75 (n=44)	0.07152
Favorable outcome (n=70)	3, 3-3.25	2, 1-2	<0.001^2^	2, 2-3 (n=31)	0.00162
Non-favorable outcome (n=47)	3, 3-4	3, 2-3	0.2502	3.5, 2-4 (n=12)	0.1252
Intramedullary tumors (n=17)	3. 2-3	2.5, 2-2.5	0.9992	2.5, 2-4 (n=10)	0.4382
Intradural extramedullary tumors (n=24)	3, 2-3	2, 2-2	0.0012	2, 2-2 (n=12)	0.03122
Extradural tumors (n=76)	3, 3-4	3, 2-3	0.0012	3, 2-4 (n=22)	0.2402

KPS, Karnofsky Performance Scale; SD, Standard Deviation; IQR, Interquartile range; ^1^Paired Student’s t-test ^2^Wilcoxon matched-pairs signed rank test. *Last follow-up 180 (51-1080) days after discharge.

At the last follow-up, these findings were further reflected by an improved long-term mMcC score only in the favorable outcome group (3 at admission vs. 2 at follow-up, p=0.0016). Interestingly, patients with intramedullary tumors returned to baseline KPS at follow-up (63.5% to 67.0%, p=0.540) after a transient postoperative deterioration at discharge (64.5% vs. 59.4%, p=0.168), without a marked improvement in their follow-up mMcC score. In patients with intradural extramedullary tumors, the mean KPS improved from 74.6±8.30% at admission to 77.5±13.9% at discharge (p=0.380) and further rose to 85.0±9.10% at follow-up (p<0.001). Accordingly, this anatomical tumor compartment group also showed an improved median mMcC score at last follow-up (3 at admission vs. 2 at follow-up, p=0.001, Wilcoxon matched pairs signed rank test). Also, patients with extradural metastases experienced a significant postoperative improvement of their mean KPS (41.6% at admission to 53.6% at discharge, p<0.001) and mMcC score (3 (3-4) vs. 3 (2-3), p<0.001) which remained stable until the last follow-up (KPS: 54.4%, p<0.061, mMcC score: 3 (2-4, p=0.240, both vs. admission).

## Discussion

There is a wealth of neurological and surgical data on patients with spinal oncologic conditions (21, 25–27), yet seldom do studies report on all different oncologic etiologies of acute paraparesis. While these patients appear to present with similar symptoms, the difference in their neurological outcome is very intriguing. In this study, we examined patients with intramedullary, intradural extramedullary, and extradural lesions and subsequent paraparesis in terms of postoperative neurological outcome and peri-operative complications. We found, that beside clinical factors such as pre-operative motor deficits, bladder, and bowel dysfunction, along with the patients’ KPS score, the different anatomical compartments of tumor localization are associated with the postoperative and long-term outcome: While patients with intradural extramedullary tumors benefit the most from surgery, the postoperative improvements seen in patients with extradural lesions patients could be off set in the long run by their progressive disease. At the same time, patients with intramedullary tumors who transiently deteriorate after surgery still show neurological improvement in the long-term.

When examining therapy objectives across all patients, alleviating their neurological deficits under a relatable surgical and medical complication risk would be crucial for maintaining an acceptable patient general condition, potentially improving survival and rendering them amenable for further chemo- and radiotherapy (28). In this context, the role of decompression and/or surgical resection in restoring and preserving neurological function after paraparesis secondary to space-occupying lesions of the spine has been confirmed in many studies (16, 23, 29, 30). Yet, and as this and previous studies show, the degree of neurological recovery of affected patients is heterogenous (31, 32). To address this rift, many efforts have been directed at determining clinical variables that predict neurological outcome after surgery, like using radiological parameters including the cord compression ratio to estimate return of ambulatory function (33). This is intended to aid prudent surgical decision-making by weighing-out potential benefits of decompression/debulking surgery and potential instrumentation against the multimorbidity-driven complications these procedures may inflict upon subsequently bed-ridden patients.

The initial analysis of this study comparing patients with ‘favorable’ vs. ‘non-favorable’ outcomes shed light not only on the importance of a preserved general condition reflected by a high KPS, which in essence is associated with pre-operative neurology, including intact bowel and bladder function, but also how vital the anatomical compartment of the spinal tumor at hand is for a postoperative recovery. While patients with intradural extramedullary lesions showed mostly favorable outcomes. This confirms previous reports on the excellent outcome of the resection of intradural extramedullary tumors, even on higher cervical levels or at an advanced age (34, 35). At the same time, the young, less-morbid patients with intramedullary tumors were less likely to recover directly after resection. And although patients with extradural metastases showed a promising postoperative recovery, they eventually succumbed to their progressive disease, underlining the importance of achieving a local long term tumor control (32). Nevertheless, the fact that most patients with restored postoperative ambulatory status (based on mMcC recovery) are from the MSCC group emphasizes the importance of early decompression surgery in such patients.

Interestingly, intradural tumors in this study became clinically apparent mostly due to gait abnormalities, while extradural metastases became symptomatic with more manifest motor deficits. Accordingly, and in line with the consensus on the importance of early decompression in preserving neurological function, the mean time from admission until surgery for MSCC patients in this study was significantly shorter than for the other two compartments. Nevertheless, patients with intradural extramedullary lesions seem to still improve after resection surgery even when it is performed after more than two days. Whether ultra-early surgery could help restore even more function in this group remains unclear.

Possible factors discussed for conferring a favorable outcome in patients with oncologic paraparesis include the use of corticosteroids and the application of electrophysiological neuromonitoring. In this cohort, corticosteroids did not deliver a better outcome for patients with oncologic paraparesis. The observation made that patients receiving corticosteroids were overrepresented in the non-favorable outcome group might, however, be associated the overrepresentation of patients with intramedullary and extradural tumors in this group, hinting to a possible confounder effect. Of note, such patients still showed worse outcomes, which is why this finding cannot be directly interpreted as an association of corticosteroids-usage with worse outcome. Nevertheless, concerns have been raised about their effectiveness vs. safety profile in patients with extradural tumors, especially when high doses are used (36, 37). The data presented on the use of electrophysiological neuromonitoring only offer limited room for interpretation, due to the low number of applicable patients. This might be explained by a tendency of surgeons to not use NM in patients with paraparesis in general, by the overrepresentation of patients with extradural metastases in the current cohort and by the often urgently and thus nightly performed surgeries with reduced availability of reliable neuromonitoring. Nevertheless, the intraoperative preservation of MEPs has been shown to predict functional outcome and hence intraoperative neuromonitoring has become a standard cornerstone in the surgery of intramedullary tumors (38, 39).

Surgical complications occurred in less than 10% of cases in the presented patient cohort and needed operative intervention in even less patients (7%). These rates appear to be lower than or comparable to what has been reported in the literature, even in cohorts containing less ‘neurologically-ill’ patients with mere intradural tumors (40–42). The fact that spinal epidural hematomas were the most common complication with two of the four postoperative bleedings occurring in the extradural metastasis group could raise concerns regarding malignancy-related coagulopathy as a cause. In contrast, analyses of patients receiving extradural decompression have shown that even under anti-coagulation, the intraoperative bleeding risk appears to be low and does e.g., not justify delaying surgery (43, 44). Interestingly, the medical complication rate in this study was significantly higher in patients with unfavorable outcomes, particularly in the presence of intramedullary tumors. This effect could be influenced by tumor location since most cervical tumors were intramedullary and cervical tumors have been previously shown to have a higher rate of pulmonary complications (45). At the same time, considering the higher proportion of ASA 3 (and higher) patients in the non-favorable outcome group, the overall medical complication risk seems to be tolerable, and scores well compared to previously published cohorts (46–48).

This study still has limitations. Firstly, its retrospective nature should be noted. Second, neurological outcome of three different paraparesis etiologies were compared using the mMcC score, which was originally developed for intramedullary lesions. Nevertheless, other scores, like the FG, have also been included in the descriptive analyses and highly correspond to outcomes found with the mMcC. Of note, radiological data evaluating the stability of the vertebral column or spinal cord compression, especially in patients with extradural metastases were not included in the analysis and could skew outcomes as well. Furthermore, defining patients with unimproved postoperative neurology and a mMcC score of 3 as a non-favorable outcome could be seen as a too strict threshold, especially with some patients still showing a theoretical ability to walk (49). However, the presented KPS data suggests that such patients are in reduced general condition compared to their counterparts with favorable outcomes. Under the criteria of this analysis, patients still qualified for a favorable outcome, even if they showed a postoperative mMcC score of 3, provided they experienced a postoperative recovery. This is because the improved postoperative neurological status offers grounds for speculation that these patients might improve further in the future. However, this assumption has not been reflected by the presented follow-up data, at least when it comes to general outcomes, showing a constant KPS in patients with a favorable outcome. In this context, further exploring of, for example, patient-reported outcomes could prove worthwhile in providing a more ‘holistic’ approach of evaluating recovery after surgery of such lesions (50, 51).

Although intra- and extramedullary tumors and extradural metastases of different primary tumors are included in this analysis, no data on “multicompartmental” or rare tumors are presented that would not fall under this strict classification. Nevertheless, examples of previous reports on spinal oncologic paraparesis in the literature include an intradural extramedullary angiosarcoma (52), an intradural extramedullary tanycytic ependymoma (usually observed intradurally) (53), and a cervical intra- and extramedullary hemangioblastoma (54). Interestingly, in all those cases, the postoperative neurological condition of involved patients mimicked our extramedullary cohort, regardless of tumor histology.

Of the three different etiologies, intramedullary tumors showed an (expected) post-operative deterioration, which has also been observed in a relevant proportion of patients with such lesions in previous reports. This could explain why the KPS score in such patients had improved markedly on the final follow-up in this analysis (25, 55). It should, however, be noted that post-discharge follow-up data in patients with intramedullary tumors mostly stems from less aggressive lesions (9 of 12 patients) who are possibly more likely to survive and show up for long-term follow-ups. Nevertheless, only 50% of the patients in this study were available for long-term follow-up which, along with missing survival data, could potentially undermine the reliability of the findings in exploring long-term outcomes. Even under this premise, a subgroup of patients with higher neurological deficits and intramedullary tumors still seem to benefit, at least in part, from surgical intervention. This could justify discussing a surgical resection in such patients even when presenting with higher motor deficits, especially if a benign tumor is suspected in radiological imaging.

In summary, patients with paraparesis secondary to spinal tumors with intramedullary localization and/or severe motor deficits reflected by a high mMcC score, a low KPS score, or an impairment of bladder and bowel functions demonstrate rather non-favorable postoperative neurological outcomes, even under the peri-operative use of corticosteroids. While patients with intramedullary tumors show mild long-term improvement upon resection or decompression, patients with extradural metastases tend to experience their best recovery postoperatively with moderate long-term improvement. However, across all different etiologies of paraparesis, even those with intramedullary lesions, patients neurologically improve after surgery. This finding enforces the role of surgery in improving the neurological function of all patients, potentially making them more amenable for adjuvant therapy. The data presented could aid a pro-surgical clinical decision-making, particularly where benefits of neurological recovery might outweigh risks of surgical intervention. At the same time, this study informs on clinical factors that might confer less-favorable postoperative neurological outcomes in patients with acute spinal oncologic paraparesis.

## Data availability statement

The raw data supporting the conclusions of this article will be made available by the authors, without undue reservation.

## Ethics statement

The studies involving human participants were reviewed and approved by Local Ethics Committee of the University of Heidelberg Medical Faculty. Written informed consent for participation was not required for this study in accordance with the national legislation and the institutional requirements.

## Author contributions

Study concept and design: AY, OTH. Data collection and analysis: SH, VL, OTH, AY. Data interpretation: OTH, AY, AU. Writing the manuscript: OTH, AY. Reviewing and editing: SH, VL, BI, KK, MS, J-ON, KZ, OTH, AY, AU. Supervision: AY, AU. All authors contributed to the article and approved the submitted version.
